# Pink esthetic treatment of gingival recession, black triangle, and gummy smile: a narrative review

**DOI:** 10.1186/s40902-025-00467-8

**Published:** 2025-07-17

**Authors:** Hyunkyung Kim, Sungtae Kim, Young-Dan Cho

**Affiliations:** Department of Periodontology, School of Dentistry and Dental Research Institute, Seoul National University and Seoul National University Dental Hospital, Seoul, Republic of Korea

**Keywords:** Gingival recession, Dental papilla, Collagen matrix, Hyaluronic acid, Botulinum toxins

## Abstract

**Background:**

With the increasing demand for comprehensive smile esthetics, pink esthetics—referring to the harmonious appearance of the gingival tissues—has gained significant attention. However, conditions such as gingival recession, black triangles, and gummy smiles can compromise these outcomes and remain challenging to manage with conventional surgical approaches. This study aimed to review minimally invasive and simplified approaches for pink esthetic enhancement using biomaterials such as collagen matrix, hyaluronic acid dermal fillers, and botulinum toxin.

**Main text:**

The use of a collagen matrix for gingival phenotype modification has demonstrated effectiveness in achieving root coverage and increasing gingival thickness while offering a less invasive alternative to traditional surgical techniques. Interdental papilla loss—commonly referred to as the “black triangle”—remains difficult to correct using both surgical and restorative procedures; however, hyaluronic acid dermal fillers offer a promising solution for reconstructing interdental gingival architecture. Additionally, excessive gingival display (gummy smile) caused by hyperactivity of the upper lip elevator muscles can be efficiently managed with botulinum toxin injections, providing a nonsurgical option for improving smile esthetics.

**Conclusions:**

The use of these biomaterials in pink esthetic management enables clinicians to achieve favorable esthetic outcomes with reduced invasiveness. This approach minimizes the need for additional restorative or surgical interventions, thereby enhancing patient comfort and satisfaction.

## Background

With rising socioeconomic standards and increasing demand for facial esthetics, concepts from facial cosmetic treatments—such as symmetry, balance, and soft tissue harmony—are being increasingly applied in dentistry [1]. Orthodontic treatment significantly contributes to establishing a well-aligned and esthetically pleasing dentition [2]. However, as patients’ esthetic expectations continue to evolve, the focus has expanded beyond tooth alignment to include the surrounding soft tissues such as the gingiva and mucosa [3]. These expanded demands have led to the emergence of “pink esthetics,” a concept that highlights the harmonious integration of the pink components (gingiva and mucosa) with the white component (teeth) [4]. While white esthetics refers to the shape, color, and restoration of teeth with suitable materials, pink esthetics refers to the health, contour, and volume of the gingiva, which plays a crucial role in achieving a balanced and attractive smile.

Among the key soft tissue factors that compromise pink esthetics are localized gingival recession (GR), black triangles resulting from loss of interdental papilla, and excessive gingival or mucosal display during smiling—commonly referred to as a gummy smile [5, 6]. Management of these conditions often requires an interdisciplinary approach to achieve high pink esthetic demands. Even at a young age, solving esthetic problems arising from pathological or functional problems is necessary, and the clinical need for GR or gummy smile is frequent among young people.

The growing interest in pink esthetics has paralleled significant advancements in biomaterials, enabling clinicians to offer less invasive treatment options for soft tissue enhancement. Collagen matrices now allow for soft tissue augmentation without the need for autogenous subepithelial connective tissue grafts, thereby reducing patient morbidity and improving acceptance [7]. Materials commonly used in facial esthetics, such as dermal fillers [8, 9] and botulinum toxin type A (commonly known by the trade name Botox) [10, 11], are increasingly being applied in dental field to enhance esthetics. As a result, patients who require expertise from facial plastic surgeons to improve their facial beauty may benefit from dental interventions that address both oral rehabilitation and improved smile. Although these treatments have gained popularity, scientific literature lacks comprehensive studies evaluating their long-term efficacy, comparative effectiveness, and potential advantages over conventional surgical approaches.

This narrative review aims to summarize current evidence on biomaterial-based interventions—including collagen matrix (CM), hyaluronic acid (HA) dermal fillers, and botulinum toxin—for the management of pink esthetic concerns such as gingival recession, interdental papilla loss, and gummy smiles. Relevant literature was identified through a non-systematic search of electronic databases, including PubMed and Google Scholar, using keywords such as “pink esthetics,” “gingival recession,” “black triangle,” “gummy smile,” “collagen matrix,” “hyaluronic acid,” and “botulinum toxin.” Studies published between 2008 and 2025 were considered. Priority was given to clinical studies, reviews, and case reports that addressed minimally invasive pink esthetic treatments involving biomaterials.

### Periodontium (Fig. 1)

**Fig. 1:**
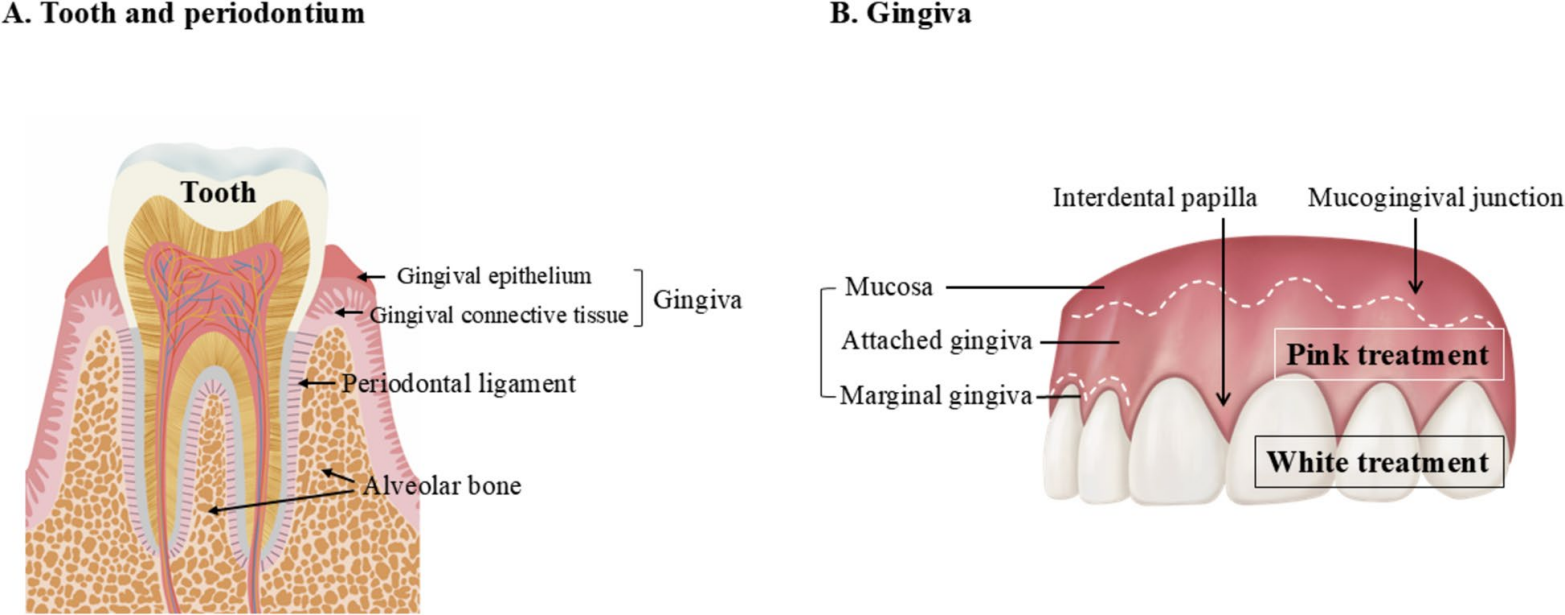
Anatomy of the periodontium. A Tooth and periodontium. B Gingiva

**Fig. 2:**
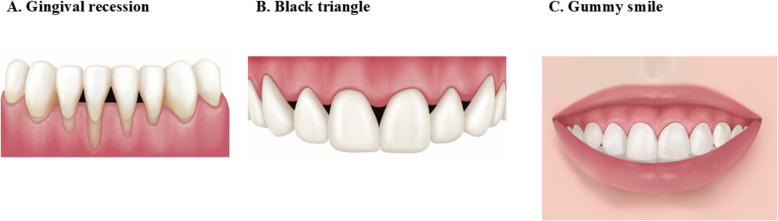
Clinical conditions requiring pink esthetics. **A** Gingival recession. **B** Black triangle. **C** Gummy smile

The periodontium is a specialized and complex structure that supports and surrounds the teeth, including soft (gingiva and periodontal ligament) and hard (cementum and alveolar bone) tissues [12] (Fig. 1A). Each component has a different location, architecture, and biochemical properties that adapt to the life of the structure. The gingiva is largely classified into two layers of epithelium and connective tissue and is composed of the marginal gingiva, attached gingiva, and mucosa, according to location (Fig. 1B). The interdental papilla is a marginal gingiva that fills the space coronal to the alveolar crest between the contact points of the teeth [13]. GR is highly likely to occur in the thin gingival phenotype when alveolar bone resorption is present (Fig. 2A) [5]. When the contact point is absent or the basal alveolar bone is resorbed, the interdental papilla migrates apically, inducing a black triangle (Fig. 2B) [14].

### Pink esthetics treatment

#### Gingival recession (Fig. 2A)

Gingival recession (GR) is defined as an apical shift of the gingival margin below the cementoenamel junction (CEJ), resulting in tooth root exposure caused by gingival loss accompanying alveolar bone resorption [15]. GR can induce unesthetic smiles, root sensitivity, and poor oral hygiene, which are related to the quality of life [16]. GR occurs frequently after orthodontic treatment; however, no clear evidence exists that orthodontic treatment alone induces GR [17]. Other causes, such as chronic periodontitis, long-lasting trauma, predisposing factors, including thin alveolar bone or gingival thickness, and high-attachment frenum insertion, could have a complicated effect. Miller’s classification has been used worldwide and is categorized into four types, depending on the relationship between the gingival margin and mucogingival junction (MGJ) and soft or hard tissue loss in the proximal area. No interdental tissue loss occurred in Miller classes I and II. In class I, the gingival margin does not extend to the MGJ; however, in class II, the gingival margin extends to or beyond the MGJ. The measurement of hard or soft tissue loss in the interproximal area is used to differentiate between classes III and IV, according to Miller’s classification. In class III, slight interdental tissue loss occurs; however, in class IV, the interdental tissue loss is severe [18]. According to Miller, complete coverage could be achieved in classes I and II; however, it was partial in class III and impossible in class IV [19].

While Miller’s classification has been widely used, it is often considered too broad and lacks the specificity to capture key clinical variables [20]. To address this limitation, the 2018 World Workshop emphasized the need for a more detailed classification system that incorporates not only the interproximal recession type but also the gingival phenotype and root surface characteristics [21, 22]. The gingival phenotype includes gingival thickness and keratinized tissue width, but underlying bone morphology should be also considered [5, 23]. Evaluating the alveolar bone with the naked eye is difficult; therefore, the gingival phenotype is mainly used in clinical settings. Additionally, alveolar bone resorption may be a fundamental problem in GR; however, most cases encounter challenges in regenerating bone and restoring its original structure. Therefore, the main treatment is soft tissue augmentation, referred to as gingival phenotype modification (GPM) [24]. GPM, covering the root surface, with a subepithelial connective tissue graft (sCTG) or free gingival graft (FGG) from the palate has been widely studied. The clinical outcomes of sCTG or FGG can be excellent; however, they create an additional surgical site for grafting and cause severe pain at the palatal donor site. Additionally, it requires skills for the detachment and attachment of gingival tissue; therefore, it requires more time and effort than the other methods. Recently, collagen matrix (CM) products have been developed to facilitate GPM procedures easily and simply [25, 26]. The use of CM reduces surgical time and provides root coverage outcomes comparable to those achieved with sCTG. A summary of the relevant studies is provided in Table 1.

**Table 1:** Summary of studies and outcomes on gingival phenotype modification with collagen matrix (CM) products

Author, year	Miller class	CM product name	F/U (months)	Mean root coverage (%)
Aroca et al., 2013 [5]	Multiple class I & II	Mucograft^a^	12	71.0
Moreira et al., 2016 [22]	Single class I & II	Mucograft^a^	6	77.2
McGuire et al., 2016 [23]	Single class I & II	Mucograft^a^	5 years	77.6
Barakat et al., 2020 [24]	Single class I & II	Mucograft^a^	12	94.2
Nahas et al., 2020 [25]	Single class I & II	Mucograft^a^	12	77.8
Stefanini et al., 2020 [26]	Single class I & II	Fibro-Gide^b^	6	96.7
Lee et al., 2021 [21]	Single/multiple class I	Collagen Graft^c^	14.5	97.0
Lakshmi et al., 2023 [27]	Multiple class I & II	Mucograft^a^	6	79.0
Harris et al., 2024 [28]	Multiple class I & II	Fibro-Gide^b^	12	Comparable results with sCTG

*CM*, xenogeneic collagen matrix; *F/U*, follow-up period; *sCTG*, subepithelial connective tissue graft. ^a^Mucograft, Geistlich Pharma AG, Wolhusen, Switzerland. ^b^Fibro-Gide. Geistlich Pharma AG, Wolhusen, Switzerland. ^c^Collagen Graft, GENOSS, Suwon, Korea

**Fig. 3:**
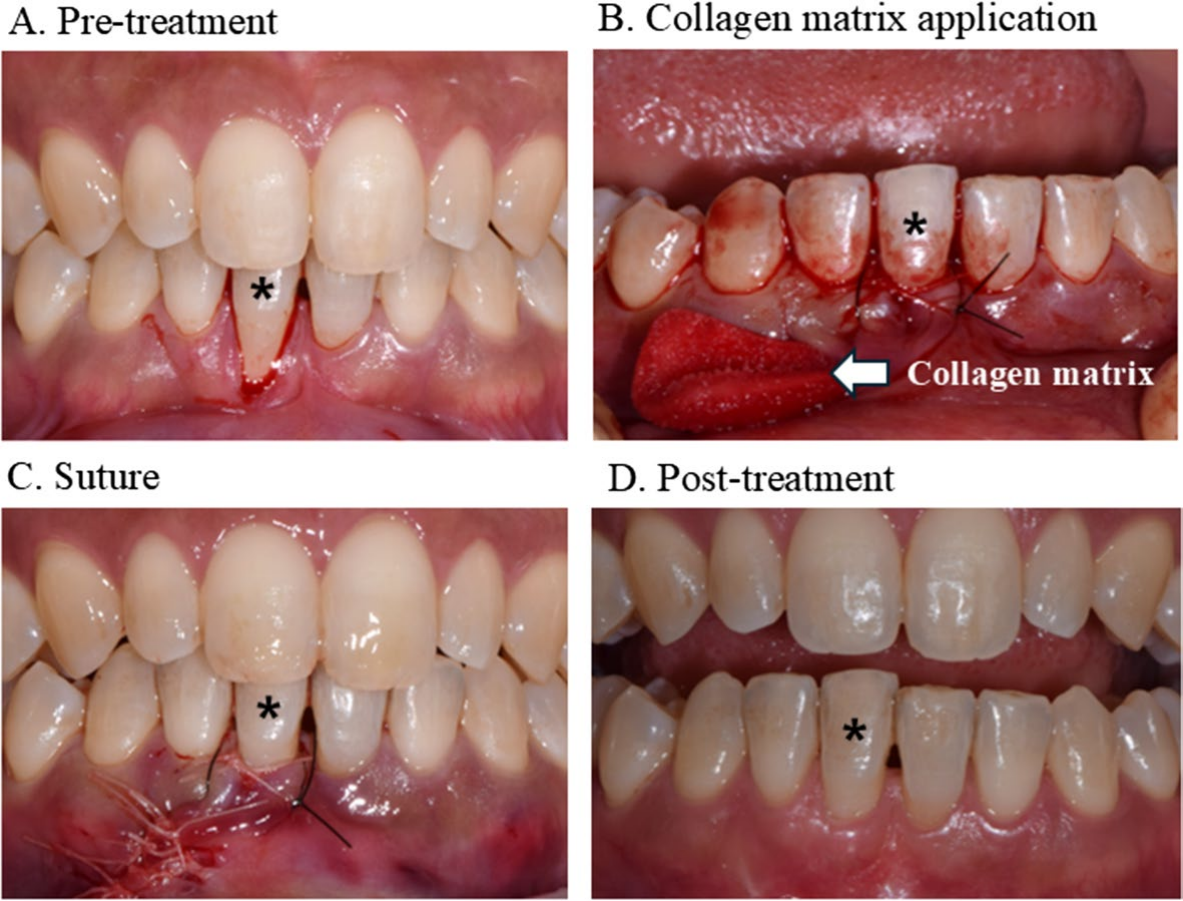
Gingival recession treatment via gingival phenotype modification. **A** Pre-treatment: Gingival recession is observed on the mandibular incisor (marked with an asterisk), classified as Miller class II. **B** Collagen matrix application: A modified tunneling technique with one vertical incision is performed, and a folded collagen matrix is inserted through the incision. **C** Suturing: Non-absorbable sutures are used for sling sutures, whereas absorbable sutures are used for the vertical incision. **D** Post-treatment: At the 1-year follow-up, successful root coverage and increased keratinized tissue thickness are observed

- Clinical case and treatment
  A 35-year-old woman visited our clinic for esthetic treatment of the mandibular lower region after orthodontic treatment. Buccal bone dehiscence occurred, and the gingival thickness was so thin that the teeth appeared transparent in the lower anterior region. Severe GR of Miller classification III was observed in tooth no. 31 (Fig. 3A). To cover the exposed root and thicken the gingiva, GPM was performed using a modified tunneling technique with a CM (Collagen Graft 2^®^, GENOSS, Suwon, Korea) (Figure 3B). At the 1-year follow-up, root coverage was well maintained, with improvements in gingival thickness and keratinized tissue width (Fig. 3C). However, the high attached frenum remained due to insufficient surgical release, which should be considered in future treatment planning.

#### Black triangle (Fig. 2B)

The black triangle is also called an open gingival embrasure at the interdental papilla and does not refer to a gap between teeth, which is a diastema [34]. However, it implies a space where teeth are connected and formed along the gingival line, which widens as they reach the root, creating a triangular shape. Simple gaps between teeth can be corrected by orthodontic treatment by aligning the teeth side by side or restoration [35]; however, the black triangle should be considered a pink esthetic treatment first because it originates from gingival volume reduction in the interproximal region. Reconstruction of the lost interdental papilla is a less predictable and challenging condition; therefore, preserving papillary integrity during dental treatment and minimizing loss as much as possible are crucial [36]. Nordland and Tarnow proposed a classification system for the loss of papillary height using three references, the interdental contact point, interproximal CEJ, and facial CEJ, and classified them into the following four classes [37]:

1. Normal: Interdental papilla fills embrasure space to the apical extent of the interdental contact point.
2. Class I: The tip of the interdental papilla lies between the interdental contact point and the most coronal extent of the interproximal CEJ.
3. Class II: The tip of the interdental papilla lies at or apical to the apical extent of the facial CEJ.
4. Class III: The tip of the interdental papilla lies at the level of or apical to the facial CEJ.

Black triangles can be induced by various factors, including aging, gingival inflammation, alveolar bone loss, and GR [13]. In addition to esthetic aspects, food packing, spitting, and phonetic problems are reasons why patients want to close their interdental embrasure [38]. When a black triangle occurs, periodontal health should first be considered. Untreated periodontal disease can be embedded under gingiva. Then white or pink esthetics treatment modalities should be selected. Nonsurgical treatment strategies for interdental papillary loss, with the injection of HA filler into the connective tissue, have recently been suggested [39]. Numerous types of HA dermal fillers are commercially available, and multiple studies have evaluated their effectiveness in reconstructing papillae and reducing the black triangles [40]. Table 2 summarizes the studies and their findings.

**Table 2:** Summary of studies and outcomes on black triangle treatment with hyaluronic acid (HA) dermal filler

Author, year	HA dermal filler product name	F/U (months)	Papilla reconstruction rate (%)
Becker et al., 2010 [30]	Unquoted	6–25	91.1
Lee et al., 2016 [31]	Teosyal PureSense Global Action VR, Teoxane, Geneva, Switzerland	6	92.6
Çankaya et al., 2020 [32]	Hyadent BG, BioScience, Germany	24	79.0
			**Reduction rate in BTA (%)**
Awartani et al., 2016 [33]	Hyadent BG, BioScience, Germany	6	41.0
Abdeloraouf et al., 2019 [34]	Restylane-Lidocaine cross-linked Hyaluronic Acid Filler, Galderma S.A., Sweden	6	45.0
			**Reduction in BTH (mm)**
Patil et al., 2020 [6]	Unquoted	3	0.85
Alhabashneh et al., 2020 [35]	Hyadent BG, BioScience, Germany	6	0.62
Pitale et al., 2021 [36]	MONALISA, GENOSS Co. Ltd., Gyeonggi R&DB Center 1 F, Suwon-si Yeongtong-gu, Gyeonggi-do, Korea	6	1.06
Ni et al., 2021 [37]	Qi Sheng Biological Agent Company Limited, Shanghai, China	12	0.28

*F/U*, follow-up period; *BTA*, black triangle area; *BTH*, black triangle height

**Fig. 4:**
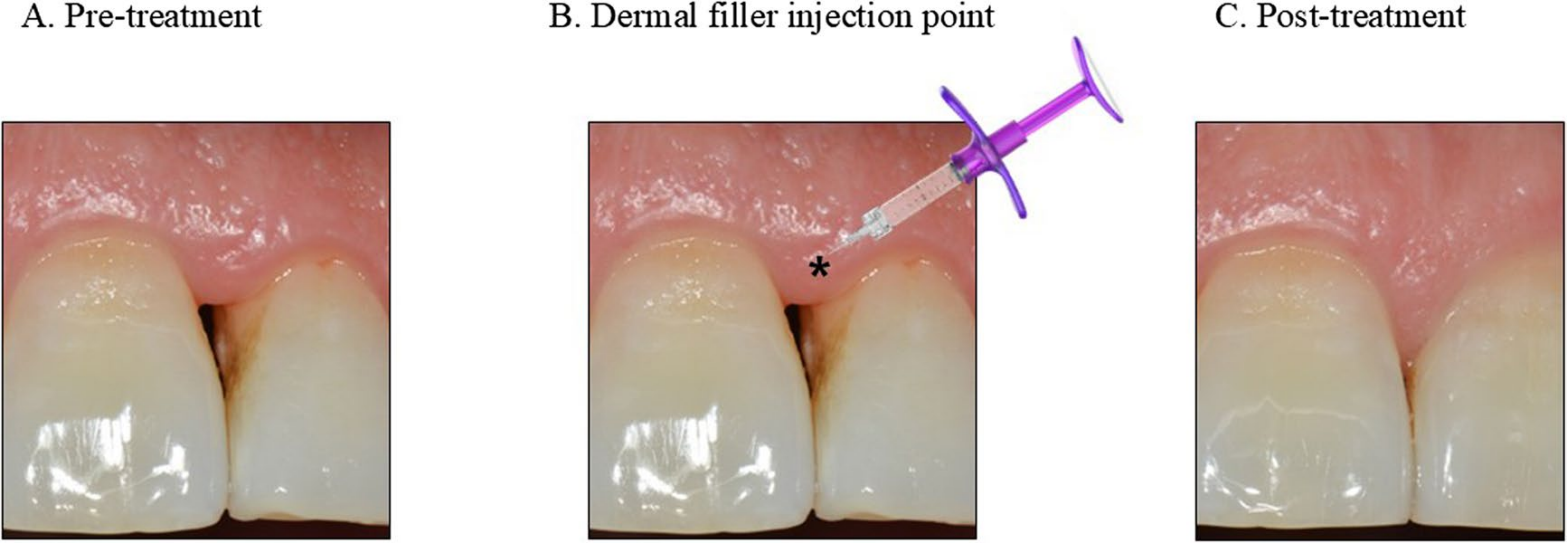
Black triangle treatment via dermal filler application. **A** Pre-treatment: The upper incisor exhibited interdental papilla loss, classified as Tarnow’s class II. **B** Dermal filler injection point: A single injection is administered 2–3-mm apical to the interdental papilla. **C** Post-treatment: Papilla fill is achieved, and the site is reclassified as Tarnow’s class I

- Clinical case and treatment
  A 57-year-old woman wanted to resolve the black triangle in the maxillary anterior region between teeth no. 21 and no. 22, which was diagnosed as Tarnow’s class II loss of the interdental papilla (Fig. 4A) [37]. Gingival inflammation with plaque and calculus deposition was observed; therefore, periodontal treatment, including scaling and root planing, was performed. After periodontal treatment, gingival inflammation was relieved, and an HA dermal filler (MONALISA, GENOSS, Suwon, Korea) was injected into the interdental gingiva (Fig. 4B). The needle was inserted at a 45° angle with the bevel directed toward the black triangle. A single-point injection was administered 2–3 mm apical to the papilla. The volume of the interproximal gingiva increased immediately after a single injection, effectively converting the site from a class II to a class I black triangle by filling the deficient space (Fig. 4C).

#### Gummy smile (Fig. 2C)

A gummy smile, known as an excessive gingival display during smiling, shows the gingiva under the upper lip [48], which is mainly a subjective clinical observation rather than a cephalometric measurement. This can be caused by excessive vertical growth of the maxilla, gingival enlargement, abnormal dental eruption, or hyperactive upper lip elevator muscles. Several factors are involved in this combination; therefore, a combined treatment is proposed to reduce gingival exposure and enhance the smile. Orthognathic surgery, orthodontic treatment, gingivectomy, and mucogingival surgery with or without ostectomy, according to the condition of the bone and keratinized tissue, are recommended for the diagnosis of skeletal problems, tooth eruption or alignment problems, gingival enlargement, and altered passive eruption, respectively [49]. To address the hyperfunction of upper lip elevator muscles, Botox injection has been suggested. The mechanism of action of Botox involves temporary paralysis or relaxation of hyperactive muscles, which excessively elevate the upper lip during a smile [50]. Table 3 provides a summary of the studies evaluating the effectiveness of Botox in alleviating gummy smiles, including the dosage used and the follow-up period.

**Table 3:** Summary of studies and outcomes of gummy smile treatment with Botox

Author, year	Product	Units (U)/point	F/U (weeks)	Improvement
Polo et al., 2008 [43]	Botox	5	> 24	98%
Mazzuco et al., 2010 [44]	Dysport	2.5–7.5	12–20	61–96%
Sucupira et al., 2012 [45]	Botox	1.95	> 12	84%
Singh et al., 2014 [46]	Botox	3	24	80%
Suber et al., 2014 [47]	Botox	4–6	12	85%
Al Wayli et al., 2019 [48]	Botox	1.95	24–36	6.05 mm
Cengiz et al., 2020 [49]	Botox	1.25–2.5	24	2.4–3.0 mm
Hexsel et al., 2020 [50]	Dysport	2.5–7.5	12	2.1–3.5 mm

*F/U*, follow-up period

**Fig. 5:**
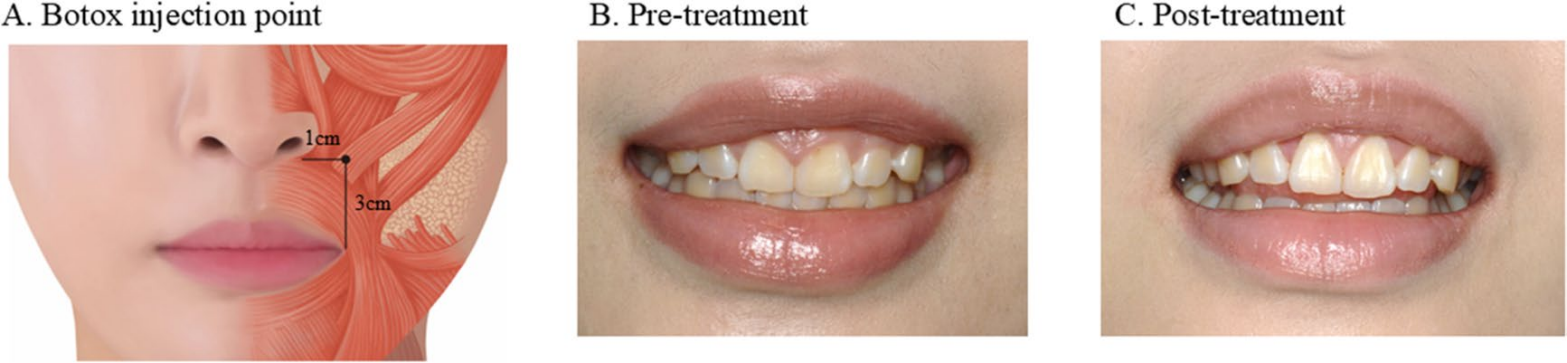
Gummy smile treatment with gingivectomy and Botox. **A** Pre-treatment: A gummy smile is observed owing to hyperactivity of the levator muscles, gingival overgrowth, and small crown height. Upper incisors are classified as type 1 A in Closet’s classification, indicating the need for a gingivectomy without an ostectomy. **B** Botox injection points: The injection site is located 1 cm horizontally from the ala of the nose and 3 cm vertically above the corner of the mouth at their intersection. **C** Post-treatment: At the 3-month follow-up, normal crown dimensions are restored, and relaxation of the upper lip elevator muscle is observed

Botox injection site, usually named the “Yonsei point,” is located 1 cm laterally to the ala of the nose horizontally and 3 cm above the oral angle vertically (Fig. 5A) [51], which is the converged region of the three main lip elevator muscles, including the levator labii superioris, levator labii superioris alaeque nasi, and zygomaticus minor. Identifying the cause and appropriate treatment options is important for the successful management of gummy smiles.

- Clinical case and treatment
  A 23-year-old woman presented with a chief complaint of a gummy smile. While smiling, the upper lip was elevated, and excessive gingival tissue covered the crown, shortening the crown length (Figure 5B). The diagnosis was a gummy smile due to hyperactive upper lips and shortened crown length caused by altered passive eruption. According to Closet’s classification, because the width of the band of keratinized tissue and the distance between the alveolar crest and CEJ were approximately 1.5 mm, we categorized it as type 1 A [52]. A gingivectomy without ostectomy was performed at the maxillary anterior teeth of nos. 13–23, to ensure normal crown length, and two units/site of Botox was injected into both Yonsei points. After 3 months of treatment, the gummy smile resolved with a proper crown length and a relaxed upper lip (Fig. 5C).

## Discussion

Esthetics has always been considered a pleasurable experience in most fields, including art, literature, food, and appearance. Dental esthetics is also a part of esthetics that focuses on smiling, dentition, and a broad region of facial esthetics. Dental esthetics are broadly classified into two parts: intraoral and extraoral. The intraoral features include white esthetics of the teeth and pink esthetics of the gingiva, whereas the extraoral features are related to the lips and skin.

For a good smile and look, various esthetic dental treatments have been performed, with white esthetic treatment receiving the most attention owing to its noninvasive and fast procedure and dramatic clinical outcomes. Additionally, with the development of digital dentistry and biomaterials, clinical outcomes can be predicted using three-dimensional devices, such as intraoral or facial scanners, to emulate their appearance, and software to simulate the digital smile design in white esthetic treatment. However, pink esthetic treatments have been recognized as difficult, time-consuming, and require skilled experience. For these reasons, white treatments have been the first choice for general dentists; however, white treatment alone has never been able to achieve 100% satisfactory results, and replacing pink may lead to unconscious or unesthetic consequences. Recent trends have returned to the basics and highlighted the importance and necessity of pink treatment using biomaterials and hints from facial esthetic treatment, which make the procedure easy and simple. The representative clinical cases of pink treatments in periodontology are GR, black triangles, and gummy smiles (Fig. 2).

Autogenous grafts, including sCTG or FGG, are considered the gold standard for soft tissue regeneration in the treatment of GR or GPM [61]. With the advantages of convenience and speed without autogenous grafting, various xenogeneic CM products from the porcine dermis, pericardium, or peritoneum are widely used; however, the pros and cons of their clinical efficacy in soft tissue augmentation remain controversial [28, 62, 63]. Long-term clinical evidence regarding the use of collagen matrices is unavailable; therefore, continuous attention and further studies are required. Injectable dermal fillers and Botox are widely used in nonsurgical cosmetic dermatology and plastic surgery. Fillers rank as the second most popular cosmetic procedure after Botox, and HA is currently the most commonly used filler material [64]. HA is a major component of connective tissue and consists of glycosaminoglycan molecules. It has anti-inflammatory effects and contributes to cell proliferation, migration, and tissue hydrodynamics by controlling the osmotic pressure. These characteristics provide stability, elasticity, and integrity to tissues and enhance tissue lubrication [65]. It is reasonable to apply dermal fillers to the mucogingival region to increase volume, as in facial esthetics, because the gingiva or oral mucosa is similar to the dermal skin in terms of its components, structures, and roles [66, 67]. Therefore, interdental gingival volume-up has been performed using dermal fillers, despite the fact that most black triangle treatments include white esthetics, including resin filling, laminate, crowns, and orthodontic treatment [35]. Botox has been widely used in neuromuscular diseases as an alternative treatment modality in the medical and dental fields, ranging from pain management to esthetics [68]. The Yonsei point could be an easy and simple site for dentists to relax the lip elevator muscles, particularly in gummy smile treatment.

Along with the cosmetic trends in dermal filler use, the number of users, including dentists, has also broadened. The use of dermal fillers or Botox for dental treatment is neither unfamiliar nor illegal; however, there is insufficient information on this topic. Different products have different degrees of effectiveness and are reversible materials with few side effects; however, periodic injections are necessary to maintain the desired clinical outcome. The increase in interest and use of pink esthetics was made possible by advances in biomaterials derived from body components, such as CM and HA. The side effects of these biomaterials are not significant; however, efforts are needed to develop better-quality materials with long-term clinical results.

## Conclusion

The increasing demand for esthetic smiles, along with the preference for less painful procedures, underscores the importance of integrating biomaterials—such as CM, HA, and Botox—into pink esthetic treatments. These approaches provide less invasive alternatives to conventional surgical techniques and can achieve favorable outcomes with relative simplicity. To fully realize these benefits, clinicians must carefully evaluate factors such as the different properties of each material, appropriate injection sites and volume, and frequency of application to optimize treatment results for individual patients. Future research should focus on comparative studies between biomaterials and traditional surgical techniques, long-term clinical outcomes, and digital workflow integration to enhance treatment predictability.

## Data Availability

No datasets were generated or analysed during the current study.

## Abbreviations

GR: Gingival recession
Botox: Botulinum toxin type A
HA: Hyaluronic acid
CEJ: Cementoenamel junction
sCTG: Subepithelial connective tissue graft
FGG: Free gingival graft
GPM: Gingival phenotype modification
CM: Collagen matrix
